# The antibacterial performance of a residual disinfectant against *Staphylococcus aureus* on environmental surfaces

**DOI:** 10.3389/fmicb.2024.1338238

**Published:** 2024-01-30

**Authors:** Soraya Omardien, Sarvesh Pingulkar, Mariska Thayagan, Laura Buniotto, Mateus de Oliveira Negreiros

**Affiliations:** ^1^Diversey Holdings Ltd., Utrecht, Netherlands; ^2^Diversey India Hygiene Pvt.Ltd., Mumbai, India; ^3^Diversey Holdings Ltd., Fort Mill, SC, United States

**Keywords:** residual disinfectant, long-lasting disinfectant, environmental surfaces, hard surfaces, *Staphylococcus aureus*

## Abstract

Environmental surfaces play a key role in transmitting pathogens that can survive on surfaces for long durations. The interest in long-lasting or residual disinfectants are, therefore, growing as it might protect surfaces for longer than traditional disinfectants. In this study, a quat-based product claiming residual disinfecting performance against bacteria, among other microorganisms, was tested using an approved standardized method, in a controlled laboratory study and on environmental surfaces in an office building. The results obtained showed that the residual disinfectant can reduce the bacterial counts significantly compared to a traditional quat-based disinfectant when used on horizontal surfaces, twenty-four hours after application. During the standardized test method, the residual disinfectant provided a 6-log reduction, whereas the traditional disinfectant provided only a 1.9-log reduction. Similarly, the residual disinfectant provided a 2.5 log reduction in the laboratory study, whereas the traditional disinfectant had too-numerous-to-count colonies. When tested on environmental surfaces, an ANOVA statistical analysis indicated that surfaces treated with the residual disinfectant had significantly less bacteria present twenty-four hours after application. The antibacterial performance of the residual disinfectant showed to be limited by the orientation of the treated surface, and the thickness of the product film dried on the surface. This study showed the potential of residual disinfectants that warrants further investigation and could potentially aid the further development of the technology.

## 1 Introduction

Environmental surfaces are known reservoirs for pathogens, and surface disinfection has shown to play an important role in reducing transmission in healthcare and public areas (Huslage et al., 2010; Wang et al., 2010; Mkrtchyan et al., 2013; Weber et al., 2013; Querido et al., 2019; Rutala and Weber, 2019). Microorganisms can survive on surfaces for long durations (Kramer et al., 2006), and pathogens can be transferred by hand after contact with these surfaces (Lopez et al., 2014; Arinder et al., 2016). When cleaning and disinfecting surfaces, the microbial load is only reduced, and re-contamination can occur rapidly (Lei et al., 2017). Therefore, there is a growing interest in long-lasting or residual disinfectants that can maintain a low microbial load on surfaces over an extended period of time.

Various disinfectants are currently emerging that claim to have residual disinfection performance, however, only one study could be found that assessed and confirmed the residual antimicrobial performance of a “continuously active disinfectant” (Rutala et al., 2019). The authors of the study confirmed a ≥ 5 log reduction of *Staphylococcus aureus* twenty-four hours after applying the product using the United States Environmental Protection Agency (EPA) approved method for residual claims (EPA 01-1A “Residual self-sanitizing activity of dried chemical residues on hard, non-porous surfaces”). In Europe, the PAS 2424 test method (PAS 2424:2014 “Quantitative surface test for the evaluation of residual antimicrobial (bactericidal and/or yeasticidal) efficacy of liquid chemical disinfectants on hard non-porous surfaces.”) is the only method available for residual disinfection claims, but so far this method has not been accepted as an official standardized method by the European Standards Organization.

In this study, we selected a quat-based product with PAS 2424 residual disinfection claims to determine whether the outcome of the product will be similar when tested using the EPA 01-1A test method. Using this standardized method, the significance of the product’s Advanced Polymer Technology in contributing to the residual performance was also assessed. The product was compared with a quat-based traditional disinfectant product without residual claims, and with a non-biocidal multipurpose cleaner. The products were further tested in a laboratory experiment and in an office building on various environmental surfaces to determine whether the residual disinfectant will outlast a traditional disinfectant in a ‘real-world’ scenario.

## 2 Materials and methods

### 2.1 Products and non-product controls selected for the study

The test products selected were Degragerm 24™ Shield ready-to-use (DG24-Shield; Diversey), Sprint Degragerm concentrate (DG-QS; Diversey), and Sprint 200 concentrate (multi-purpose cleaner; MPC; Diversey). DG24-Shield has shown to be antibacterial and antiviral by providing ≥4 log reduction of bacteria and viruses within 5 min after application using European standardized methods (EN1276, EN13697, EN14476, and EN16777). It has yeasticidal efficacy within 15 min as shown with the EN13697 and has residual efficacy claims against bacteria and enveloped viruses using PAS 2424. The DG24-Shield contains 0.25% w/v alkyl (C12-16) dimethyl benzyl ammonium chloride (ADBAC) with 0.25% w/v didecyl dimethyl ammonium chloride (DDAC) and has a pH between 3.5 and 4.6. The DG24-Shield formulation without the Advanced Polymer Technology was also tested to confirm the importance of the technology for its long-lasting performance. DG-QS was diluted to 0.5% v/v in demineralized water to obtain a ready-to-use product concentration. The diluted product has antimicrobial efficacy claims against bacteria, yeast, fungi and enveloped viruses. It contains 0.1% w/v ADBAC and 0.009% w/v N,N-bis (3-aminopropyl) dodecylamine (APDA) with a pH ranging from 10.5 to 11.4. The MPC has no antimicrobial efficacy claims and was diluted to 8% v/v using demineralized water. The dilutions were selected based on the prescribed in-use concentration of each product available in the product information sheet. During the laboratory study, sterile demineralized water was included as a non-product control. All the products selected were sprayed 15 times onto the wipe before applying it to the test surface. The spraying frequency was selected based on the amount that was needed to sufficiently pre-wet or moisten the wipe with the water control used in the laboratory test. Taski Allegro wipes (Diversey) were used, which is a non-woven cloth consisting of 85% viscose and 15% polypropylene.

### 2.2 EPA residual disinfection test method to assess antimicrobial performance after applying abrasions

The residual disinfectant claims of the DG24-Shield were confirmed using the EPA 01-1A “Residual self-sanitizing activity of dried chemical residues on hard, non-porous surfaces” (RSD) method [United States Environmental Protection Agency (EPA), 2023a]. The method was followed as recommended, but with the exception that the product was applied and allowed to dry on the surface for 30 min before applying the initial inoculum. Typically, the method requires that the initial inoculum is applied onto the test surface before applying the test products to determine the initial antimicrobial performance of the product. A schematic of the steps taken during performing the EPA 01-1A method can be found in Supplementary Figure S1. The test culture selected was *S. aureus* strain ATCC® 6538 as it has been prescribed for testing sanitizers and disinfectants^1^ and can be transmitted through hands (Solberg, 2000).

The first cycle included both dry and wet abrasions. Dry abrasions involved wiping the surface with a dry cloth using a Gardner apparatus. The surface was re-inoculated with the test culture, containing a final concentration of 5% w/w organic soiling, and allowed to dry for 30 min. Wet abrasions were subsequently performed by wiping the surface with a wet cloth using the same Garner apparatus (Gardco). The cycle ends by re-inoculating the test culture onto the test surface after completing the wet abrasion step. The dry and wet abrasion cycle were performed six times within 24 h. The survival of the test culture re-inoculated onto the test surface after the final cycle was quantified and should result in a three-log reduction compared to the untreated control for claiming residual sanitization, or a five-log reduction when claiming residual disinfection, according to the EPA efficacy protocols for bacteria [United States Environmental Protection Agency (EPA), 2023b]. The contact time, organic soiling and carriers used in all cases were 10 min, 5% w/v fetal bovine serum (FBS; Cytiva Hycone) and stainless steel (McMaster-Carr), respectively. The products were tested twice, with each repeat consisting of three experimental repeats.

### 2.3 Preparation of the culture used during the laboratory study

The culture selected for determining the immediate antibacterial efficacy of the products was *S. aureus* strain ATCC® 6538. *S. aureus* was prepared by streaking the culture onto tryptic soy agar (TSA; Oxoid) from a freeze culture stock and incubating the agar plate for 24 h at 37°C. The latter was used to transfer an actively growing culture, using an inoculation loop, to 5 mL tryptic soy broth (TSB; Oxoid) in a sterile Falcon tube. After incubating the culture for 24 h at 37°C, the entire 5 mL culture in TSB was transferred to 1 L of TSB. The culture was incubated for 24 h at 37°C and diluted with diluent to have about 1.5 to 5.0 × 10^8^ CFU/mL. The latter diluted culture was used to apply the bacteria onto the test surface before applying the test product. This culture was used to determine immediate antimicrobial efficacy. The culture was also used to re-inoculate the test surfaces after applying the products (wet abrasion; refer to Section 2.4.2). The sterile diluent contained 0.1% w/v tryptone pancreatic digest of casein (Oxoid) and 0.85% w/v sodium chloride (Supelco) dissolved with demineralized water to have a final volume of 1 L. The diluent prepared had a final pH of 7.0 ± 0.2 and was autoclaved. From the 24 h culture, 2.5 mL was transferred to 500 mL of TSB, and incubated again for 24 h at 37°C. The 24 h culture was diluted with diluent to have about 1.5 to 5.0 ×10^8^ colony forming units per milliliters (CFU/mL).

### 2.4 Immediate efficacy and antimicrobial performance after 24 h

#### 2.4.1 Preparation of the test surfaces

To evaluate the immediate antibacterial efficacy of the selected products and their performance after 24 h, the products were applied onto the top surface of a laboratory work bench. The surface was divided into 15 × 15 cm squares using general laboratory labeling tape (Supplementary Figure S2). For each product, three squares were selected as experimental repeats. A set of three squares per product or control were made for each time point. The selected swab sampling points where 5 min after the product has been applied (immediate efficacy test), and 2, 4 and 24 h after product application. Sterile cotton swabs (Herenz, Heinz GMBH) were used. Each test surface was cleaned with 70% v/v ethanol prior to starting the experiment.

#### 2.4.2 Description of the dry and wet abrasions performed

Dry abrasions were performed by wiping the test surface with a dry towel once, with a downward sweeping motion from top to bottom while applying force (Supplementary Figure S2). The towels used for dry abrasions were Taski Allegro wipes (Diversey). Wet abrasion was performed by placing a gloved hand inoculated with *S. aureus* onto the surface with a downward sweeping motion from top to bottom, while applying force. The gloved hand was inoculated with bacteria by placing it with the palm facing downwards and the fingers spread out wide into a 100 mL pre-prepared *S. aureus* culture (refer to Section 2.3). The culture was in a sterile petri-dish with a diameter of 15 cm. The gloved hand was kept in the culture for 3 s, and then allowed to drip off the fingers for 30 s while keeping the fingers spread out wide before performing the wet abrasion. Dry abrasions were performed at 2 h and 3 h, and wet abrasions at 4 h and 24 h, after applying the test products. The 2, 3, and 4 h time points were selected as it were evenly distributed within the first day of the experiment, and 24 h was selected as it is the final time point at which the technology claims antibacterial performance.

During the laboratory test, controls were included to observe the impact of the abrasions on the culture applied to the surface and for the test products, which were “no cleaning with water or products but only wet abrasions” (NC), “no cleaning but wet and dry abrasions” (NW), and “cleaning with sterile water and both wet and dry abrasions (W).” NC was included to evaluate the microbial load present when bacteria was applied but no cleaning or dry abrasions were performed. NW was included to evaluate the microbial load applied on the surface during the wet abrasions, but when no product was used to clean the surface and only wiping with a dry wipe was performed. NW was performed to determine whether the bacteria can be removed with the dry wipe alone. The W control was to evaluate the microbial load when water was used to clean the surface instead of the test products. The W control was exposed to both wet and dry abrasions.

#### 2.4.3 Applying the product, performing the abrasions, and observing the bacteria survival

Prior to cleaning the test surfaces with the selected products, the *S. aureus* culture was applied to the test surfaces and left to air dry for 30 min at room temperature (about 20°C), and with a relative humidity ranging from 56 to 70%. The *S. aureus* culture used was prepared as described in Section 2.3, and its application was similar as described for “wet abrasions” in Section 2.4.2. The survival of *S. aureus* on the test surfaces were represented by the NC control described in Section 2.4.2. The test products were then applied as specified by the product guidelines, which were to spray the product onto a wipe, to wipe the surface and to wait until the product dries on the surface. A swab sample was taken after a 5 min contact time with the applied product to evaluate immediate antibacterial efficacy. For testing long-term efficacy, the test product was left to dry on the surface and dry abrasions were performed 2 and 3 h after application. Wet abrasions were performed 4 and 24 h after application, which involved re-inoculating about 4.6 × 10^6^ bacteria onto the surface.

Dry abrasions and wet abrasions were performed as described in Section 2.4.2. Swab samples were taken 30 min after the wet abrasions were performed at 4 h and 24 h after application. The swab used for sampling was pre-wetted with a neutralizer known to quench the quat in the products. The efficacy of the neutralizer was validated during the pre-work of this study using the prescribed method in the EN 13727 “Quantitative suspension test for the evaluation of bactericidal activity in the medical area.” The neutralizer consisted of 3% w/v Tween 80 (Sigma-Aldrich), 0.3% w/v L-alpha-lecithin (Acros organics), 0.1% w/v histidine (Millipore), 0.5% w/v sodium thiosulphate (Supelco), 3% w/v saponin (VWR chemicals), and 0.0025 mL phosphate buffer and made up to 1 L with demineralized water. The swab sample was added to 10 mL diluent in a test tube and vortexed (Dilution 1). From this test tube, 1 mL of the mixture was taken and added to 9 mL diluent (Dilution 2). From both dilutions, 1 mL was taken and added to a sterile petri-dish to perform the pour-plate technique. The latter was performed in duplicate. The composition of the diluent can be found in Section 2.3. The culturing was performed with TSA and incubation was at 37°C. The plates were inspected within 24 h, followed by 48 h, and the results were reported as CFU/mL.

#### 2.4.4 Determining the concentration of the bacteria transferred to the surface

The culture transferred to the surface from an inoculated hand was estimated by placing the pre-inoculated gloved hand into a sterile petri-dish with a diameter of 15 cm. Diluent (10 mL) was added to the petri-dish containing the bacteria left from the gloved hand. Mixing was performed by swirling the solution for about 30 s. This was performed to bring the bacteria into suspension for the subsequent dilution steps. A dilution series was performed of the mixture in diluent up to 10^7^. From each dilution, 1 mL was removed and added to a petri-dish for the pour-plate technique. The latter was performed in duplicate. The culturing media was TSA, and the incubation conditions were 48 h at 37°C. Counts were performed after 24 h and again after 48 h to take into consideration smaller colonies. Results were expressed as CFU/mL and countable plates contained between 14 and 330 CFU/mL. The composition of the diluent can be found in Section 2.3.

### 2.5 Evaluating the residual efficacy of DG24-shield in an office building

#### 2.5.1 Description of the office building selected for the study

Various areas in an office building were selected for treatment and its surface area documented. The areas included vertical surfaces (door handles and touch screens) and horizontal surfaces (desks and touch screens). Vertical surfaces included ten typical door handles from six different locations, a door handle with a larger surface area, a door button touched with a flat hand palm, and two touch screens for coffee machines located at two separate locations. The horizontal surfaces included two touch screens from a printer, six desk surfaces from two different locations, and one reception desk. In total, fourteen vertical surfaces and nine horizontal surfaces were tested. The areas selected were cleaned on two separate days to signify test repeats, and swab samples were taken 24 h after application. The number of people present in the building were monitored for 4 months, and it showed to be comparable on Tuesdays, Wednesdays, and Thursdays (Supplementary Figure S3). These days were selected to perform the environmental field trial. Cleaning was performed by a laboratory staff member who was unaware of the aim of the study (“unbiased cleaner”). The general cleaning staff was informed of the study and was instructed not to clean the areas marked for this study. Information about the test surfaces selected can be found in Supplementary Table S1.

#### 2.5.2 Antibacterial efficacy of the products on environmental surfaces

Cleaning of the areas with the selected products were performed with a dry towel (Taski Allegro wipe, Diversey). The “unbiased cleaner” was instructed to apply the product to the wipe so that it was sufficiently wet according to their satisfaction, which was a squirting action of four times with the spray bottle. The amount of squirting was subsequently increased to seven times, and then ten times. The products were applied onto the test surface and a swab sample was taken 24 h after application. Each product was tested on two separate days (repeats). Environmental surfaces had to be cleaned daily, therefore the multipurpose cleaner was selected to obtain the baseline. The performance of DG-QS and DG24-Shield were compared to the multipurpose cleaner based on the presence of bacteria on the surface twenty-four hours after application.

The amount of product applied to the surfaces after wiping was quantified by weighing the product when sprayed onto the dry wipe, wiping/cleaning a 30 × 30 cm surface square and calculating the difference in weight of the wipe afterwards. The liquid release profile of the product when applied to the wipe was performed similar, but 16 squares were cleaned after spraying the product onto the wipe.

The swabs used for sampling were pre-wetted with neutralizer as described in Section 2.4.5. The swab samples were added to 1 mL neutralizer and kept for further processing directly after sampling. A dilution series was performed within 30 min after sampling. To each swab sample in the 1 mL neutralizer, 9 mL diluent was added (Dilution 1). From the first dilution, 1 mL was taken and added to 9 mL diluent (Dilution 2). From each dilution, 1 mL was removed and added to a petri-dish for the pour-plate technique. The latter was performed in duplicate. The culturing media was TSA, and the incubation conditions were 48 h at 37°C. Results were expressed as colony forming units per 100 cm^2^ (CFU/100 cm^2^).

#### 2.5.3 Statistical analysis of the data collected in the environmental field study

The data obtained during the evaluation of the test products on environmental surfaces were expressed as microbial counts (CFU/100 cm^2^) and subjected to statistical analysis. The data set was log transformed by using the “log function” to convert the skewed data set to conform to normality. Normality of the log transformed data set was confirmed using the Anderson-Darling normality test. The test of equal variances was conducted before moving forward with ANOVA statistical analysis. The ANOVA analysis was performed on the log transformed data set using the Tukey and Games-Howell test. The experiment was repeated (Repeat 1 and 2) and represent the application of the products on two separate days. Each repeat was considered as a separate data set and were treated accordingly during the statistical analysis. All statistical analyses were performed using Minitab software version 19. A confidence interval (CI) of 95% was selected as the data set consisted of a small sample size and had variations typical of environmental field trials.

## 3 Results

### 3.1 Residual self-disinfecting activity using the EPA standardized method

The EPA 01-1A test method was employed to confirm the residual disinfection claims of DG24-Shield, but also to confirm whether the Advanced Polymer Technology (APT) of the product contributes to its performance. DG24-Shield with and without the polymer technology were compared with a traditional disinfectant product, DG-QS, and with a multipurpose cleaner, Sprint 200. The results obtained can be found in Table 1.

**Table 1:** The RSD results to determine the residual performance of the test products.

Product name	Log reduction of *Staphylococcus aureus* after 24 h			
	Repeats 1	Repeats 2	Average	Standard deviation
DG24-Shield with APT^#^	5.79	6.23	6.01	0.31
DG24-Shield without APT^#^	3.32	3.13	3.23	0.13
DG-QS	1.63	2.25	1.94	0.44
MPC	1.09	1.59	1.34	0.35
	Log counts of *S. aureus* that survived 24 h after being applied			
Untreated culture control	7.15	7.55	7.35	0.28

Each repeat represents the average value of three experimental repeats. ^#^APT, Advance polymer technology.

During the execution of the test, about 7.35 log *S. aureus* remained on the surface after 24 h. The stainless-steel surface treated with DG24-Shield provided a 6.01 log reduction when compared with the untreated culture control. The residual disinfection claims of DG24-Shield were, therefore, confirmed. Treatment with the DG24-Shield formulation without APT resulted in only a 3.23 log reduction. Therefore, confirming that the technology is essential for the product’s residual performance. DG-QS and the MPC both failed the ≥5 log reduction requirement of the EPA 01-1A test method as only a 1.94 and 1.34 log reduction were obtained, respectively. Conclusively, DG24-Shield fulfilled the criteria for residual disinfection claims using the EPA 01-1A test method.

### 3.2 Antimicrobial efficacy of the products tested in a laboratory study

The immediate antibacterial performance was evaluated based on swabs taken after 5-min of product application. The estimated *S. aureus* cells applied on the workbench surface was about 3 × 10^6^ CFU/mL (Table 2). Cleaning with water showed a 3-log reduction (Table 2). Water might remove microorganisms from the surfaces when using a wipe, but it will remain on the wipe and transfer microorganisms to other surfaces (Ledwoch and Maillard, 2018). The multipurpose cleaner (MPC; Sprint 200) provided a 3.89-log reduction. The cleaner and disinfectant, DG-QS, provided a 4.89-log reduction, and DG24-Shield provided a ≥ 5-log reduction. Even though both DG-QS and DG24-Shield reduced the microbial load sufficiently, DG24-Shield was superior in performance. This was expected as DG24-Shield had a higher quat concentration than DG-QS.

**Table 2:** The results obtained in the laboratory study to determine whether the residual disinfectant (Degragerm 24™ Shield) will outperform the multipurpose cleaner (MPC) and traditional disinfectant (Degragerm QS; DG-QS) twenty-four hours after application.

Test conditions	*S. aureus* survival after treatment (CFU/ml)		Log reduction
	Mean	Standard error	
Bacterial counts 5 min after applying the products			
Starting culture	2.58 × 10^8^	8.74 × 10^6^	
Transferred by hand	3.14 × 10^6^	1.26 × 10^6^	
NC	≥ 3.14 × 10^6^	≥ 3.14 × 10^6^	
NW	≥ 3.14 × 10^6^	≥ 3.14 × 10^6^	
W	2.63 × 10^3^	3.24 × 10^3^	3.08
MPC	4.04 × 10^2^	5.73 × 10^2^	3.89
DG-QS	4.00 × 10^1^	6.93 × 10^1^	4.89
DG24-Shield	0.00 × 10^0^	0.00 × 10^0^	≥ 5
Bacterial counts after wet abrasions were performed 4 h after applying the products			
Starting culture	2.92 × 10^8^	1.17 × 10^8^	
Transferred by hand	6.54 × 10^6^	2.60 × 10^5^	
NC	≥ 6.54 × 10^6^	≥ 6.54 × 10^6^	
NW	≥ 6.54 × 10^6^	≥ 6.54 × 10^6^	
W	≥ 6.54 × 10^6^	≥ 6.54 × 10^6^	< 1
MPC	2.26 × 10^5^	3.03 × 10^5^	1.46
DG-QS	7.91 × 10^3^	5.40 × 10^3^	2.92
DG24-Shield	1.03 × 10^3^	1.62 × 10^3^	3.8
Bacterial counts after wet abrasions were performed 24 h after applying the products			
Starting culture	1.44 × 10^8^	3.30 × 10^7^	
Transferred by hand	2.66 × 10^6^	1.35 × 10^6^	
NC	≥ 2.66 × 10^6^	≥ 2.66 × 10^6^	
NW	≥ 2.66 × 10^6^	≥ 2.66 × 10^6^	
W	≥ 2.66 × 10^6^	≥ 2.66 × 10^6^	< 1
MPC	≥ 2.66 × 10^6^	≥ 2.66 × 10^6^	< 1
DG-QS	≥ 2.66 × 10^6^	≥ 2.66 × 10^6^	< 1
DG24-Shield	8.36 × 10^3^	1.19 × 10^4^	2.5

The other test conditions included: no cleaning with products but wet abrasions (NC), no cleaning but wet and dry abrasions (NW), and cleaning with sterile water and both wet and dry abrasions (W). The mean represents the results of three biological repeats.

Long term performance was evaluated after 4 h. The surfaces treated with water had *S. aureus* counts higher than what could be counted and was reported to have <1 log reduction. The surface treated with the MPC, DG-QS, and DG24-Shield had a 1.46, 2.92, and 3.80 log reduction, respectively. DG-QS showed some antibacterial efficacy after 4 h on the surface, which might be because of the quat present. Quat is known to remain on the surface for a long period of time (Burel et al., 2021), however, it is unclear for how long. Conclusively, DG24-Shield was the best performing product 4 h after application.

Twenty-four hours after the products were applied only one product showed efficacy. DG24-Shield demonstrated residual antibacterial efficacy by reducing the bacterial counts by 2.5 logs. The surfaces cleaned with water, the MPC and DG-QS had bacterial counts that were uncountable (< 1 log reduction).

### 3.3 Antimicrobial efficacy of the products tested on environmental surface in an office building

In this study, we aimed to understand whether DG24-Shield would outperform DG-QS and the MPC (Sprint 200) in reducing the microbial load of ‘real-world’ environmental surfaces 24 h after application. The baseline for the environmental field trial was determined by spraying the multipurpose cleaner (MPC) four times onto the wipe before cleaning the surface. DG-QS and DG24-Shield were applied in a similar manner. The two products (DG-QS and DG24-Shield) were compared with the baseline (MPC), and both the liquid release profile (Supplementary Figure S4) and amount of product applied to the surface was similar between the test products (Supplementary Table S2). Swab sampling was performed 24 h after applying the product, and the maximum counts obtained for the MPC was about 7 × 10^3^ CFU/100 cm^2^.

The data obtained from the swab sampling 24 h after applying the products was subjected to a “Normality test” and was observed to be non-normal. The “log function” was used to convert the data (Supplementary Tables S3–S5) to a log transformed data set (Table 3). Subsequently, the “test of equal variances” was employed to determine the appropriate significance method to be used along with the ANOVA analysis. The “test of equal variances” for the vertical surfaces demonstrated equal variance with a 95% confidence interval (CI), therefore the “Tukey method” was employed. However, the null hypothesis for horizontal surfaces was rejected and an unequal variance was observed, which required the use of the “Games-Howell method.”

**Table 3:** The log transformed data set used for the ANOVA statistical analysis.

Environmental surfaces	MPC		DG-QS		DG24-Shield		DG-QS		DG24-Shield		DG-QS		DG24-Shield	
	4x		4x		4x		7x		7x		10x		10x	
	R1	R2	R1	R2	R1	R2	R1	R2	R1	R2	R1	R2	R1	R2
Vertical surfaces														
Door handle 1	2.2	2.5	3.0	2.4	2.3	2.4	3.0	2.2	2.2	2.6	2.8	2.0	0.9	2.1
Door handle 2	3.2	2.4	2.6	2.8	2.7	2.2	2.3	2.1	2.6	2.3	3.1	4.2	3.4	2.7
Door handle 3	3.2	3.2	2.6	2.9	2.4	2.7	3.4	3.1	2.7	3.0	3.3	3.4	2.5	2.5
Door handle 4	3.4	3.7	3.4	3.5	1.8	3.1	3.8	3.4	3.5	3.6	3.8	3.7	3.2	3.2
Door handle 5	3.2	2.5	2.7	2.9	2.6	2.9	3.1	3.0	1.8	2.2	2.6	2.8	2.4	2.0
Door handle 6	3.8	3.7	3.2	3.3	3.8	3.0	3.8	3.8	3.1	2.3	3.2	3.1	3.6	3.1
Door handle 7	3.4	3.2	2.9	2.4	2.5	2.4	3.0	2.6	2.5	2.4	3.7	3.3	2.4	2.8
Door handle 8	4.0	3.2	3.0	3.2	2.7	3.0	3.2	3.1	2.6	3.2	4.5	3.3	3.2	2.6
Door handle 9	3.9	3.6	3.9	3.6	3.9	3.8	4.2	3.9	3.3	3.2	3.6	3.4	4.8	3.4
Door handle 10	3.7	2.2	3.1	3.1	2.4	2.8	2.7	2.3	2.7	3.0	3.6	2.4	3.0	2.7
Door handle	1.8	1.9	1.9	2.0	2.8	1.5	1.7	2.6	1.2	1.5	1.4	1.9	2.2	0.6
Door button	2.4	2.2	3.0	3.9	3.1	2.3	2.0	1.5	2.0	1.6	2.0	2.6	2.5	2.9
Coffee machine 1	3.5	2.1	2.8	2.7	2.4	2.7	1.7	1.9	1.4	0.4	2.2	2.5	1.8	0.4
Coffee machine 2	3.3	2.0	2.6	2.3	2.9	2.7	2.8	2.4	1.7	2.5	2.6	2.3	0.9	1.4
Mean	3.21	2.74	2.91	2.92	2.73	2.69	2.90	2.71	2.37	2.42	3.03	2.93	2.62	2.31
Std dev	0.66	0.66	0.46	0.55	0.57	0.53	0.77	0.70	0.70	0.83	0.82	0.67	1.04	0.92
R1 R2 Combined mean	2.98		2.92		2.71		2.81		2.39		2.98		2.47	
R1 R2 Combined std dev	0.66		0.51		0.55		0.74		0.77		0.74		0.98	
Horizontal surfaces														
Printer 1	1.7	1.4	2.1	2.1	1.0	1.7	1.8	1.8	0.0	1.4	1.1	1.3	0.0	0.3
Printer 2	2.5	2.4	2.7	2.9	2.1	1.9	2.3	1.4	1.9	1.4	2.4	2.7	0.9	1.7
Table 1	2.9	2.6	3.6	2.3	1.0	2.0	2.4	2.3	1.7	1.3	1.5	2.5	0.8	0.3
Table 2	2.8	2.4	3.6	3.4	2.0	1.7	2.1	1.9	1.3	1.6	2.5	2.1	0.3	0.0
Table 3	3.4	2.3	3.3	3.5	1.3	1.4	2.5	2.3	2.1	1.4	1.9	1.8	0.3	0.0
Table 4	1.5	2.5	2.7	2.9	0.6	1.8	2.7	1.5	1.2	1.7	1.9	3.3	0.3	0.3
Table 5	2.6	2.7	2.6	3.0	3.7	2.9	2.3	2.6	2.0	2.2	2.7	2.5	2.0	2.1
Table 6	1.9	2.3	2.3	3.0	2.5	2.7	2.0	2.6	2.0	2.3	2.4	3.2	2.3	2.4
Reception desk	3.4	3.5	2.7	2.4	2.9	2.5	2.2	2.0	1.6	1.9	2.0	2.0	1.8	1.6
Mean	2.51	2.45	2.85	2.84	1.90	2.07	2.27	2.05	1.54	1.67	2.02	2.39	0.98	0.97
Std dev	0.70	0.53	0.56	0.48	1.00	0.50	0.29	0.44	0.65	0.38	0.54	0.65	0.84	0.97
R1 R2 Combined mean	2.48		2.85		1.98		2.16		1.60		2.21		0.98	
R1 R2 Combined std dev	0.62		0.52		0.75		0.36		0.52		0.60		0.90	

The data represents the bacteria counts present on the test surfaces 24 h after cleaning and disinfection.

The ANOVA analysis performed on the log transformed data set from vertical surfaces showed a value of *p* of 0.05 (Total degrees of freedom of 13 and a *F*-value of 1.80), which is equal to the 0.05 criteria required for a significant difference at a 95% confidence level. The ANOVA analysis graphs can be found in Supplementary Figure S5. The Tukey method was employed to determine which data set showed a statistically significant difference. No separate grouping was obtained for the different data sets, which suggests that there was no statistical difference between the test conditions for vertical surfaces (Table 4). DG24-Shield, therefore, performs similarly to the MPC and DG-QS when applied to vertical surfaces.

**Table 4:** The grouping comparison of the vertical surfaces using the Tukey method with a 95% confidence interval.

Factor	Number	Mean	Grouping
Sprint 200 (4x) Repeat 1	14	3.212	A
DG-QS (10x) Repeat 1	14	3.030	A
DG-QS (10x) Repeat 2	14	2.927	A
DG-QS (4x) Repeat 2	14	2.925	A
DG-QS (4x) Repeat 1	14	2.906	A
DG-QS (7x) Repeat 1	14	2.903	A
Sprint 200 (4x) Repeat 2	14	2.741	A
DG24-Shield (4x) Repeat 1	14	2.728	A
DG-QS (7x) Repeat 2	14	2.709	A
DG24-Shield (4x) Repeat 2	14	2.689	A
DG24-Shield (10x) Repeat 1	14	2.624	A
DG24-Shield (7x) Repeat 2	14	2.416	A
DG24-Shield (7x) Repeat 1	14	2.371	A
DG24-Shield (10x) Repeat 2	14	2.310	A

The means that do not share a letter are significantly different.

The ANOVA analysis performed on the log transformed data set from horizontal surfaces showed a significant difference as a value of *p* of 0.00 (Total degrees of freedom of 13 and a F-value of 7.67) was obtained. In contrast with vertical surfaces, the Games-Howell method showed that DG24-Shield when applied seven or ten times formed a separate grouping compared to the other test products for both repeats (Table 5). The statistical analysis, therefore, confirmed that DG24-Shield outperformed the other test products when applied to horizontal surfaces.

**Table 5:** The grouping comparison of the horizontal surfaces using the Games-Howell method with a 95% confidence interval.

Factor	Number	Mean	Grouping		
DG-QS (4x) Repeat 1	9	2.853	A		
DG-QS (4x) Repeat 2	9	2.844	A		
Sprint 200 (4x) Repeat 1	9	2.513	A	B	
Sprint 200 (4x) Repeat 2	9	2.451	A	B	
DG-QS (10x) Repeat 2	9	2.388	A	B	
DG-QS (7x) Repeat 1	9	2.2674	A	B	
DG24-Shield (4x) Repeat 2	9	2.074	A	B	
DG-QS (7x) Repeat 2	9	2.052	A	B	
DG-QS (10x) Repeat 1	9	2.025	A	B	
DG24-Shield (4x) Repeat 1	9	1.896	A	B	C
DG24-Shield (7x) Repeat 2	9	1.669		B	C
DG24-Shield (7x) Repeat 1	9	1.535		B	C
DG24-Shield (10x) Repeat 1	9	0.980			C
DG24-Shield (10x) Repeat 2	9	0.972			C

The means that do not share a letter are significantly different.

## 4 Discussion

Microorganisms are known to survive for long durations on inanimate surfaces (Kramer et al., 2006) and it is known that environmental surfaces play an important role in transmitting pathogens (Suleyman et al., 2018). Knowledge is limited concerning the performance of long-lasting or residual disinfectants even though there is an interest in the technology. Only two studies were found, where one was an environmental field trial conducted in a healthcare environment (Schmidt et al., 2019) and the other was a laboratory study using an EPA method for residual claims (Rutala et al., 2019). Only the environmental field trial study concluded that the long-lasting or residual disinfectant can outlast the other traditional disinfectants tested.

In this study, the residual disinfection capabilities of a product called Degragerm 24™ Shield (DG24-Shield) were evaluated. It was formulated with an Advanced Polymer Technology (APT) that forms a film on the surface when it dries. This dried polymer film physically entraps the biocide (quat) in-between its network, enabling the biocide to easily diffuse to the surface to interact with bacteria or viruses. Simultaneously, the APT film forms a protective layer that prevents or reduced the removal of the biocide during physical abrasions. Currently, the DG24-Shield has residual disinfection claims against bacteria and enveloped viruses using the non-standardized PAS 2424 test method. The US EPA method (EPA 01-1A) for residual claims was employed in this study to confirm the performance of DG24-Shield, but also to confirm the importance of the polymer for the product’s long-lasting effect. The results obtained confirmed the residual performance of DG24-Shield and the importance of the polymer in protecting the biocide from multiple abrasion cycles.

Even though the product met the criteria of the EPA test method for residual claims, it was still unclear whether the product will outlast traditional disinfectants in performance twenty-four hours after application. Environmental surfaces are challenging areas to test disinfectants as various uncontrolled situations can occur, such as the incorrect application of the product or the spillage of soil on the test surface. To understand whether DG24-Shield will outperform traditional disinfectants in a ‘real-world’ scenario, a laboratory test and an environmental field trial in an office building was performed. The laboratory test simulated an environmental field trial, but the product was applied onto the surfaces and monitored in a controlled manner. The application of the products in the environmental field trial, however, were uncontrolled and reflects the ‘real-world’ situation. In both cases, DG24-Shield outperformed the multipurpose cleaner and quat-based disinfectant (DG-QS) as less bacteria were recovered from treated surfaces twenty-four hours after application. However, performance could only be confirmed for horizontal surfaces. The results obtained for vertical surfaces, which were predominantly door handles, were not significantly different.

The surface area treated with the product and the area sampled might have had an impact on the outcome of the results obtained for vertical surfaces. Of the fourteen vertical surfaces selected, ten were door handles. Nine door handles had a surface area of 60 cm^2^, and one had a surface area of 75 cm ^2^. Door handles are touched with the entire hand palm and potentially at a higher frequency throughout the day. The entire area of the door handle was also sampled to evaluate the bacteria present, which was about 100% of the treated surface. However, the surface area of the horizontal surfaces is larger and only a small area is sampled. For instance, one treated table had a treated surface area of 10,920 cm^2^, but the sampling area was only 225 cm^2^. This means only 2% of the total area were sampled at a time. Another reason for the difference in performance observed between vertical and horizontal surfaces could be that the product does not dry at the thickness required to have the long-lasting effect. When DG24-Shield was applied four times to the test surfaces, no difference was observed compared to the other test products, but when more product was applied a difference was observed. This highlights that the amount of product applied has an impact on the performance of the product. Thus far, the test surfaces used in the PAS 2424 test method, the EPA 01-1A test method, the laboratory test and the environmental field trial where DG24-Shield outperforms the traditional disinfectant are horizontal. Taken together, the findings highlight that the orientation of the treated surface, the sampling area verses the treatment area, the surface abrasion frequency, and the thickness of the product film formed on the surface could potentially impact the performance of long-lasting disinfection technology, which requires further investigation.

Only one study evaluated the antibacterial efficacy of a “continuously active disinfectant” using a standardized method (Rutala et al., 2019). The authors could obtain a 5-log reduction for *S. aureus*. A ≥ 4 log reduction could be obtained for vancomycin-resistant *Enterococcus*, *Escherichia coli* and *Enterobacter* spp. and only 1.5 log reduction for *Klebsiella pneumoniae*. All the CRE bacteria tested had less than 3 log reduction. A difference in performance was also observed when the product was tested against *S. aureus* on formica and stainless-steel carriers (Rutala et al., 2019). The “continuously active disinfectant” product tested contained 0.276% ADBAC, 0.104% DDAC, 0.207% octyl decyl dimethyl ammonium chloride (ODDMAC), 0.104% dioctyl dimethyl ammonium chloride (DODMAC) and 68.61% ethanol. DG24-Shield has a lower quat concentration, which consist of 0.25% w/v ADBAC with 0.25% w/v DDAC, and a comparison between the two products cannot be drawn. To understand the antibacterial performance of DG24-Shield against other pathogens or on different materials will contribute to the understanding of long-lasting disinfection technology and guide its appropriate application in use.

## 5 Conclusion

In conclusion, a residual disinfectant was evaluated to determine its antibacterial performance after 24 h in three different test methods. In all cases, the residual disinfectant outperformed the multi-purpose cleaner and a traditional disinfectant when applied to horizontal surfaces. It was observed that the amount of residual disinfectant applied to the surface had an important impact on its antibacterial performance, which should be taken in consideration when making use of such technology. Additionally, the results obtained concerning the antibacterial performance of the residual disinfectant on vertical surfaces was inconclusive. This study highlights the potential that residual or long-lasting disinfectants have, which warrants further investigation to determine its impact on the environment, whether positive or negative.

## Data availability statement

The original contributions presented in the study are included in the article/Supplementary material, further inquiries can be directed to the corresponding author.

## Author contributions

SO: Conceptualization, Data curation, Investigation, Methodology, Supervision, Writing – original draft. SP: Data curation, Writing – review & editing. MT: Data curation, Writing – review & editing. LB: Data curation, Writing – review & editing. MO: Data curation, Writing – review & editing.

## Footnotes

^1^ https://www.atcc.org/products/6538
