# Supplementary material for: The antibacterial performance of a residual disinfectant against Staphylococcus aureus on environmental surfaces
Source: Front Microbiol. 2024 Jan 30;15:1338238. doi: 10.3389/fmicb.2024.1338238 (PMC10861741; doi:10.3389/fmicb.2024.1338238)
Supplement: Supplementary file 1 [file Data_Sheet_1.docx]

Supplementary Material


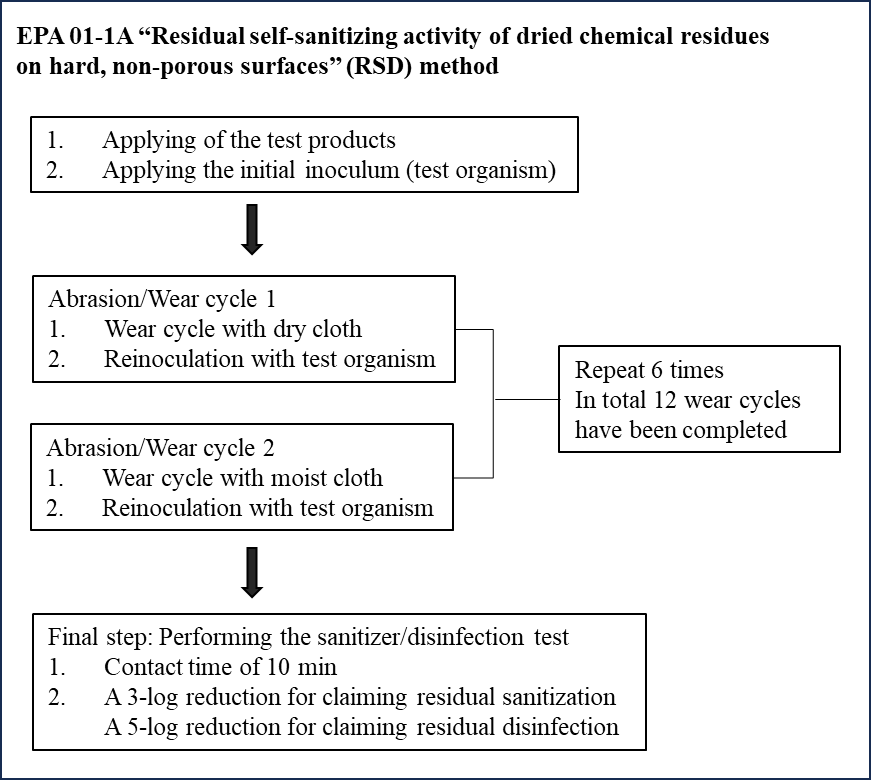


**Supplementary Figure 1.** An illustration depicting the steps taken to perform the EPA 01-1A test method for claiming residual disinfection.


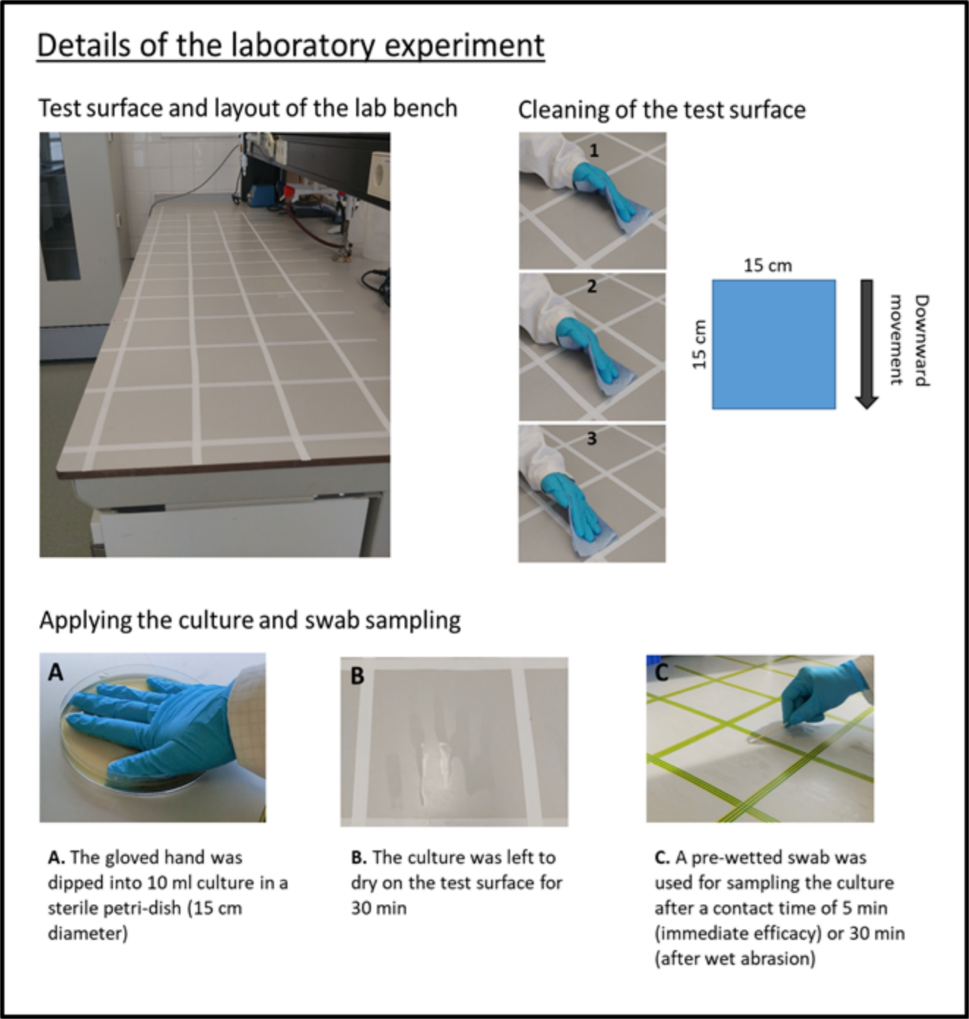


**Supplementary Figure 2.** The laboratory experiment was performed by dividing the test surface into 15 x 15 cm squares. Each test condition was performed in triplicate. Cleaning with the products was performed with a downward sweeping motion. (A) The culture was applied with a gloved hand that was pre-dipped in a *S. aureus* culture. (B) The culture was allowed to dry for 30 min on the test surface. (C) Swab sampling was performed after the initial cleaning step, and 30 min after performing the wet abrasions.


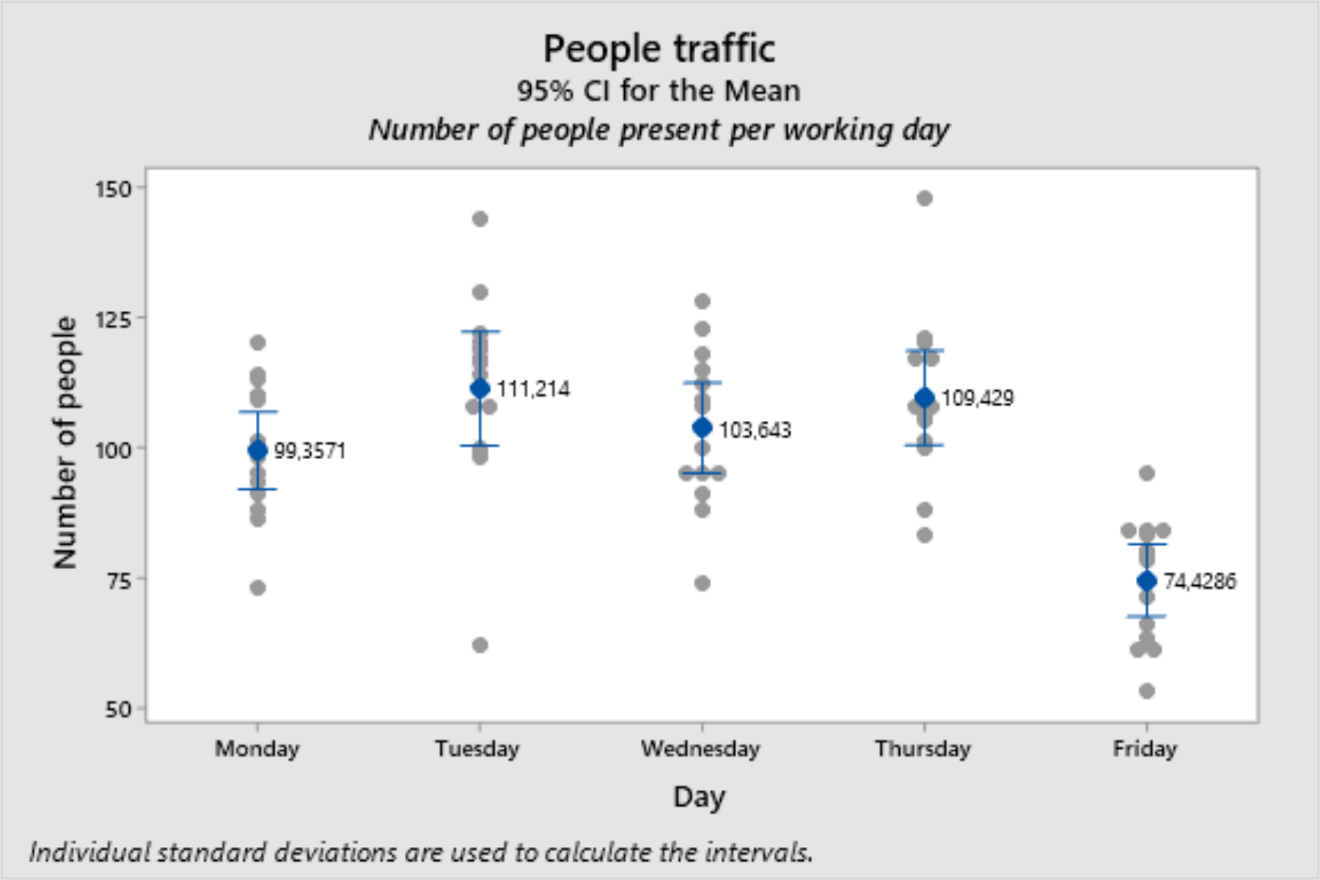


**Supplementary Figure 3.** The number of people present in the office building per day over the duration of four months. The products were applied on Tuesdays or Wednesday, and swab sampling was performed 24 hrs after applying the product.


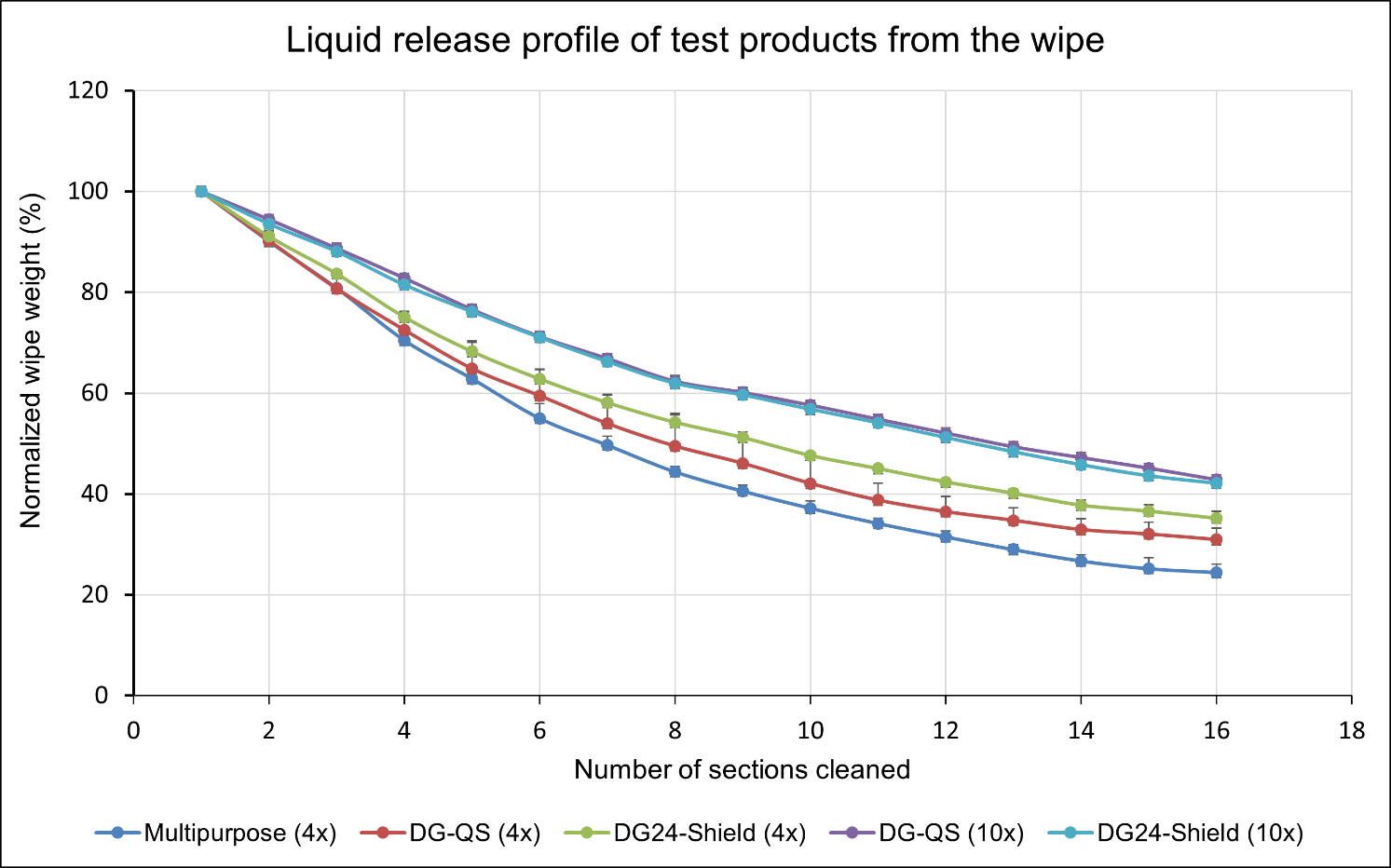


**Supplementary Figure 4.** The liquid release profile of the test products used in the environmental field trial.


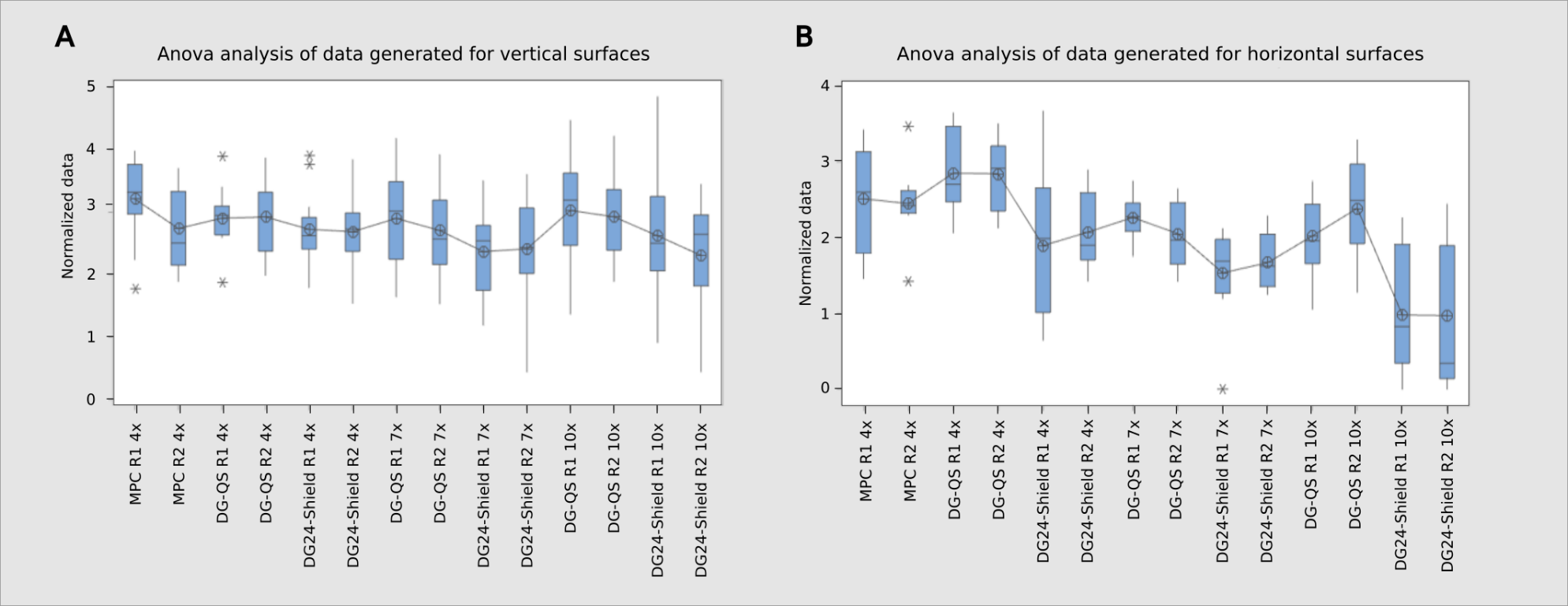


**Supplementary Figure 5.** The box charts from the ANOVA statistical analysis of the data generated during the environmental field trial for vertical (A) and horizontal (B) surfaces. The solid black line within the box depicts the median, and the upper and lower quartiles are shown as the bottom and top edges of the box. The lines that extend below and above the box have endpoints that correspond to the lowest and highest value in the data set. The circles depict the mean of the data sets.

| **Test surface** | **Touch frequency** | **Surface area (cm^2^)** |
| --- | --- | --- |
| **Vertical surfaces** | | |
| Door handle 1 | Medium | 60 |
| Door handle 2 | Medium | 60 |
| Door handle 3 | Medium | 60 |
| Door handle 4 | Medium | 60 |
| Door handle 5 | Medium | 60 |
| Door handle 6 | Medium | 60 |
| Door handle 7 | Medium | 60 |
| Door handle 8 | Medium | 60 |
| Door handle 9 | High | 60 |
| Door handle 10 | Low | 75 |
| Door handle | Low | 255 |
| Door Button | Low | 130 |
| Coffee machine 1 | Medium | 178 |
| Coffee machine 2 | Medium | 178 |
| **Horizontal surfaces** | | |
| Printer 1 | Low | 281 |
| Printer 2 | Low | 281 |
| Table 1 | Low | 225 |
| Table 2 | Low | 225 |
| Table 3 | Low | 225 |
| Table 4 | Low | 225 |
| Table 5 | Low | 225 |
| Table 6 | Low | 225 |
| Reception desk | Low | 225 |

**Supplementary Table 1.** The different test surfaces selected and additional information. The touch frequency noted in the table was estimated.

| **Test product** | ***Amount on wipe (g)*** | ***Amount after cleaning (g)*** | ***Amount left onto the surface (g)*** |
| --- | --- | --- | --- |
| ***When the product is sprayed onto the wipe 4x*** | | | |
| MPC | 4.27 ± 0.28 | 3.85 ± 0.27 | 0.42 ± 0.01 |
| DG-QS | 4.63 ± 0.49 | 4.18 ± 0.51 | 0.45 ± 0.06 |
| DG24-Shield | 4.77 ± 0.09 | 4.35 ± 0.05 | 0.43 ± 0.05 |
| ***When the product is sprayed onto the wipe 7x*** | | | |
| DG-QS | 7.91 ± 0.33 | 7.27 ± 0.40 | 0.64 ± 0.10 |
| DG24-Shield | 8.64 ± 0.14 | 8.03 ± 0.14 | 0.62 ± 0.02 |
| ***When the product is sprayed onto the wipe 10x*** | | | |
| DG-QS | 10.51 ± 0.25 | 9.73 ± 0.25 | 0.77 ± 0.02 |
| DG24-Shield | 11.82 ± 0.07 | 11.05 ± 0.09 | 0.76 ± 0.06 |

**Supplementary Table 2.** The amount of product left behind after cleaning a 30 x 30 cm desk surface. The mean represents the results of three experimental repeats.

| **Test surfaces** | **MPC (Baseline)** | | **DG-QS** | | **DG24-Shield** | |
| --- | --- | --- | --- | --- | --- | --- |
|  | **± 4 g** | **± 4 g** | **± 5 g** | **± 5 g** | **± 5 g** | **± 5 g** |
|  | **CFU/100cm^2^** | | | | | |
|  | **Repeat 1** | **Repeat 2** | **Repeat 1** | **Repeat 2** | **Repeat 1** | **Repeat 2** |
| **Vertical surfaces** | | | | | | |
| Door handle 1 | 175 | 308 | 1100 | 242 | 200 | 250 |
| Door handle 2 | 1500 | 225 | 392 | 625 | 500 | 142 |
| Door handle 3 | 1667 | 1417 | 422 | 858 | 267 | 542 |
| Door handle 4 | 2417 | 5250 | 2567 | 2833 | 58 | 1225 |
| Door handle 5 | 1750 | 333 | 458 | 833 | 358 | 875 |
| Door handle 6 | 7000 | 4833 | 1708 | 1833 | 5917 | 1083 |
| Door handle 7 | 2250 | 1750 | 775 | 233 | 300 | 258 |
| Door handle 8 | 9500 | 1667 | 1025 | 1617 | 542 | 942 |
| Door handle 9 | 7769 | 3923 | 7862 | 4231 | 8231 | 6923 |
| Door handle 10 | 5467 | 160 | 1127 | 1400 | 227 | 613 |
| Door handle | 59 | 78 | 75 | 98 | 588 | 35 |
| Door Button | 231 | 146 | 1019 | 7308 | 1200 | 200 |
| Coffee machine 1 | 2865 | 135 | 579 | 475 | 281 | 489 |
| Coffee machine 2 | 1938 | 104 | 438 | 185 | 730 | 534 |
| **Horizontal surfaces** | | | | | | |
| Printer 1 | 51 | 27 | 116 | 135 | 10 | 55 |
| Printer 2 | 303 | 265 | 519 | 814 | 133 | 80 |
| Table 1 | 778 | 356 | 4116 | 211 | 11 | 107 |
| Table 2 | 622 | 244 | 4418 | 2489 | 98 | 47 |
| Table 3 | 2378 | 200 | 2102 | 3178 | 20 | 27 |
| Table 4 | 29 | 289 | 504 | 822 | 4 | 67 |
| Table 5 | 400 | 489 | 444 | 1067 | 4689 | 778 |
| Table 6 | 76 | 222 | 200 | 1022 | 287 | 484 |
| Reception desk | 2667 | 3000 | 462 | 240 | 711 | 318 |

**Supplementary Table 3.** Bacterial counts of swab samples taken from environmental surfaces 24 hrs after treatment or application. The test products were sprayed 4 x onto the wipe before cleaning the test surfaces. The results are expressed as colony forming units (CFU) per 100 cm^2^ area. The criteria established were as follows: 0 and 36 CFU/100 cm^2^ were considered very good to good (GREEN), between 37 and 116 CFU/100 cm^2^ were considered satisfactory (YELLOW), and more than 116 CFU/100 cm^2^ was considered poor performance (RED). The criteria are set for the number of bacteria present on the surfaces.

| **Test surfaces** | **MPC (Baseline)** | | **DG-QS** | | **DG24-Shield** | |
| --- | --- | --- | --- | --- | --- | --- |
|  | **± 4 g** | **± 4 g** | **± 8 g** | **± 8 g** | **± 9 g** | **± 9 g** |
|  | **CFU/100cm^2^** | | | | | |
|  | **Repeat 1** | **Repeat 2** | **Repeat 1** | **Repeat 2** | **Repeat 1** | **Repeat 2** |
| **Vertical surfaces** | | | | | | |
| Door handle 1 | 175 | 308 | 1000 | 158 | 142 | 392 |
| Door handle 2 | 1500 | 225 | 208 | 117 | 375 | 192 |
| Door handle 3 | 1667 | 1417 | 2500 | 1333 | 458 | 1058 |
| Door handle 4 | 2417 | 5250 | 6167 | 2733 | 3000 | 4333 |
| Door handle 5 | 1750 | 333 | 1250 | 950 | 58 | 142 |
| Door handle 6 | 7000 | 4833 | 5750 | 5750 | 1333 | 200 |
| Door handle 7 | 2250 | 1750 | 1083 | 358 | 325 | 250 |
| Door handle 8 | 9500 | 1667 | 1583 | 1333 | 433 | 1667 |
| Door handle 9 | 7769 | 3923 | 15000 | 8308 | 2077 | 1615 |
| Door handle 10 | 5467 | 160 | 467 | 220 | 480 | 987 |
| Door handle | 59 | 78 | 49 | 392 | 16 | 31 |
| Door Button | 231 | 146 | 108 | 35 | 104 | 42 |
| Coffee machine 1 | 2865 | 135 | 45 | 79 | 23 | 3 |
| Coffee machine 2 | 1938 | 104 | 646 | 264 | 51 | 298 |
| **Horizontal surfaces** | | | | | | |
| Printer 1 | 51 | 27 | 57 | 61 | 0 | 23 |
| Printer 2 | 303 | 265 | 189 | 27 | 81 | 23 |
| Table 1 | 778 | 356 | 267 | 200 | 49 | 18 |
| Table 2 | 622 | 244 | 133 | 80 | 22 | 42 |
| Table 3 | 2378 | 200 | 311 | 211 | 131 | 24 |
| Table 4 | 29 | 289 | 558 | 33 | 16 | 47 |
| Table 5 | 400 | 489 | 222 | 444 | 91 | 169 |
| Table 6 | 76 | 222 | 111 | 396 | 100 | 193 |
| Reception desk | 2667 | 3000 | 156 | 93 | 40 | 73 |

**Supplementary Table 4.** Bacterial counts of swab samples taken from environmental surfaces 24 hrs after treatment or application. The test products were sprayed 7 x onto the wipe before cleaning the test surfaces. The results are expressed as colony forming units (CFU) per 100 cm^2^ area. The criteria established were as follows: 0 and 36 CFU/100 cm^2^ were considered very good to good (GREEN), between 37 and 116 CFU/100 cm^2^ were considered satisfactory (YELLOW), and more than 116 CFU/100 cm^2^ was considered poor performance (RED). The criteria are set for the number of bacteria present on the surfaces.

| **Test surfaces** | **MPC (Baseline)** | | **DG-QS** | | **DG24-Shield** | |
| --- | --- | --- | --- | --- | --- | --- |
|  | **± 4 g** | **± 4 g** | **± 11 g** | **± 11 g** | **± 12 g** | **± 12 g** |
|  | **CFU/100cm^2^** | | | | | |
|  | **Repeat 1** | **Repeat 2** | **Repeat 1** | **Repeat 2** | **Repeat 1** | **Repeat 2** |
| **Vertical surfaces** | | | | | | |
| Door handle 1 | 175 | 308 | 675 | 108 | 8 | 117 |
| Door handle 2 | 1500 | 225 | 1408 | 16417 | 2583 | 458 |
| Door handle 3 | 1667 | 1417 | 1917 | 2250 | 292 | 283 |
| Door handle 4 | 2417 | 5250 | 6500 | 4750 | 1667 | 1575 |
| Door handle 5 | 1750 | 333 | 417 | 583 | 242 | 92 |
| Door handle 6 | 7000 | 4833 | 1750 | 1250 | 3583 | 1275 |
| Door handle 7 | 2250 | 1750 | 4750 | 2083 | 267 | 625 |
| Door handle 8 | 9500 | 1667 | 29167 | 2083 | 1433 | 433 |
| Door handle 9 | 7769 | 3923 | 4077 | 2539 | 70154 | 2769 |
| Door handle 10 | 5467 | 160 | 3733 | 267 | 927 | 467 |
| Door handle | 59 | 78 | 24 | 78 | 143 | 4 |
| Door Button | 231 | 146 | 108 | 423 | 346 | 808 |
| Coffee machine 1 | 2865 | 135 | 160 | 337 | 62 | 3 |
| Coffee machine 2 | 1938 | 104 | 365 | 197 | 8 | 25 |
| **Horizontal surfaces** | | | | | | |
| Printer 1 | 51 | 27 | 11 | 19 | 0 | 2 |
| Printer 2 | 303 | 265 | 265 | 530 | 8 | 47 |
| Table 1 | 778 | 356 | 29 | 333 | 7 | 2 |
| Table 2 | 622 | 244 | 287 | 133 | 2 | 0 |
| Table 3 | 2378 | 200 | 76 | 62 | 2 | 0 |
| Table 4 | 29 | 289 | 71 | 1956 | 2 | 2 |
| Table 5 | 400 | 489 | 556 | 311 | 107 | 131 |
| Table 6 | 76 | 222 | 244 | 1667 | 182 | 278 |
| Reception desk | 2667 | 3000 | 91 | 111 | 62 | 36 |

**Supplementary Table 5.** Bacterial counts of swab samples taken from environmental surfaces 24 hrs after treatment or application. The test products were sprayed 10 x onto the wipe before cleaning the test surfaces. The results are expressed as colony forming units (CFU) per 100 cm^2^ area. The criteria established were as follows: 0 and 36 CFU/100 cm^2^ were considered very good to good (GREEN), between 37 and 116 CFU/100 cm^2^ were considered satisfactory (YELLOW), and more than 116 CFU/100 cm^2^ was considered poor performance (RED). The criteria are set for the number of bacteria present on the surfaces.
